# Somatostatin analog therapy in delaying progression of polycystic liver disease: A meta-analysis with trial sequential analysis

**DOI:** 10.1016/j.iliver.2026.100220

**Published:** 2026-01-16

**Authors:** Mohammed S. Beshr, Rana H. Shembesh, Bisher Sawaf, Shahem Abbarh, Mohammed Abu-Rumaileh, Monica Tincopa, Muhammed Elhadi

**Affiliations:** a Department of Medicine, Faculty of Medicine and Health Sciences, Sana'a University, Sana'a, Yemen; b Department of Medicine, Faculty of Medicine, Libyan International Medical University, Benghazi 999116, Libya; c Department of Internal Medicine, University of Toledo Medical Center, Toledo, OH 43614, USA; d Department of Internal Medicine, MedStar Health, Georgetown University, Baltimore, MD 21218, USA; e MASLD Research Center, Division of Gastroenterology & Hepatology, Department of Medicine, University of California, San Diego, CA 92037, USA; f Department of Medicine, College of Medicine, Korea University, Anam-ro, Seongbuk-gu, Seoul 02841, Republic of Korea

**Keywords:** Somatostatin, Polycystic liver disease, Autosomal dominant polycystic kidney disease

## Abstract

**Background and aims:**

Polycystic liver disease (PLD) can occur independently or in association with autosomal dominant polycystic kidney disease and has no effective medical therapy. This meta-analysis evaluated whether somatostatin analog therapy slows progression of PLD.

**Methods:**

The PubMed, Scopus, Web of Science, and Cochrane databases were searched through to May 1, 2025 to identify randomized controlled trials that evaluated somatostatin analogs in PLD. The primary endpoints were percentage change from baseline in total liver volume (TLV), total kidney volume (TKV), and estimated glomerular filtration rate (eGFR). Safety endpoints included cholelithiasis, cholecystitis, diarrhea, and abdominal pain. Effect sizes were estimated using mean differences (MDs) and odds ratios (ORs) within a random-effects framework. Trial sequential analysis was performed for TLV, TKV, and eGFR.

**Results:**

Seven randomized controlled trials with 640 participants were included. Somatostatin analog therapy led to a significant reduction in TLV (MD −6.73%, 95% CI −8.68 to −4.78; *p* ​< ​0.001; GRADE, low)]and TKV (MD −3.35%, 95% CI −4.97 to −1.74; *p* ​< ​0.001; GRADE, moderate) compared with placebo. No significant effect was observed for eGFR (MD 0.32, 95% CI −4.55 to 5.19; *p* ​= ​0.90; GRADE, low). Adverse events, including cholelithiasis and cholecystitis (OR 4.66, 95% CI 1.19–18.26; *p* ​= ​0.03; GRADE, moderate), diarrhea, and abdominal pain, were more frequent in the somatostatin group. Trial sequential analysis showed good evidence of benefit for TLV and TKV and inconclusive evidence for eGFR.

**Conclusions:**

Based on low-to-moderate evidence, somatostatin analogs were significantly associated with reduced TLV and TKV, but not eGFR, in patients with PLD, and with higher adverse event rates. Long-term trials are warranted to define their role in clinical management.

**Registration**: PROSPERO, ID CRD420251045402.

## List of abbreviations

PLD: polycystic liver disease
ADPKD: autosomal dominant polycystic kidney disease
cAMP: cyclic adenosine monophosphate
eGFR: estimated glomerular filtration rate
TLV: total liver volume
TKV: total kidney volume

## 1 Introduction

Polycystic liver disease (PLD) is defined by the presence of 20 or more cysts in the liver.^1^ It occurs most often in association with autosomal dominant polycystic kidney disease (ADPKD), with hepatic cysts seen in over 90% of individuals with ADPKD over the age of 35 years.^2^^,^^3^ However, PLD can also occur as an isolated condition known as autosomal dominant PLD, which is much rarer, affecting less than 1 in 10,000 individuals (less than 0.01% of the US population).^4^

Most patients with isolated PLD are asymptomatic, and the condition is often diagnosed incidentally on imaging.^5^ Among individuals with a substantial cyst burden, there is potential for significant clinical symptoms and a risk of development of portal hypertension, varices, jaundice, ascites, and a Budd–Chiari-like syndrome.^5^^,^^6^

ADPKD has a prevalence of 3 per 10,000 population, accounting for 5%–10% of cases of kidney failure in both the USA and Europe.^7^^,^^8^

To date, there is no treatment that effectively delays the progression of PLD or ADPKD. Current management of PLD is largely supportive, focusing on symptom control, and cyst aspiration, with surgical intervention for severe cases.^5^

Elevated cyclic adenosine monophosphate (cAMP) levels have been implicated in the pathogenesis of PLD and ADPKD, contributing to rapid cyst growth, increased cell proliferation, and progressive tissue damage.^9^ Therefore, lowering cAMP levels is a logical therapeutic goal.

Somatostatin analogs can lower cAMP levels and have emerged as a therapeutic option for PLD and ADPKD.^10^ In experimental models of these diseases, long-acting release (LAR) somatostatin analogs such as octreotide have been shown to reduce cAMP levels and slow disease progression by targeting somatostatin receptors.^11^^,^^12^ These analogs bind to receptors on cystic cholangiocytes, suppressing release of cAMP, reducing production of cyst fluid, and inhibiting bile duct cell hyperplasia, all of which may help to prevent proliferation of hepatic cysts.^1^

Trials examining these agents have shown promise in the treatment of cysts in the kidney and liver.^13^^,^^14^ This systematic review and meta-analysis evaluated the efficacy of somatostatin analog therapy in PLD. Its effects on other outcomes, including total kidney volume (TKV), estimated glomerular filtration rate (eGFR), and safety outcomes, were also assessed.

## 2 Materials and methods

The study was performed in accordance with the 2020 Preferred Reporting Items for Systematic Reviews and Meta-Analyses (PRISMA) standards.^15^ The PRISMA checklist for this meta-analysis is found in the Supplementary Table S1. The protocol's registration number is CRD420251045402.

### 2.1 Literature search

The PubMed, Scopus, Web of Science, and Cochrane databases were searched through to May 1, 2025. The strategy combined Medical Subject Headings and keywords related to “somatostatin analogs” and “autosomal dominant polycystic kidney disease” or “autosomal dominant polycystic liver disease”, using Boolean operators (AND, OR) to structure the queries. No language, region, or time restrictions were applied. In addition to searching the databases, we performed cited reference tracking and manually screened the bibliographies of the included studies to identify additional relevant articles. Supplementary Table S2 describes our search strategy and the terms used in more detail.

### 2.2 Study selection

Randomized controlled trials (RCTs) that evaluated the effectiveness of somatostatin analogs in slowing the progression of kidney or liver disease were eligible for inclusion. There were no restrictions related to language, sex of participants, or geographic region. The following exclusion criteria were applied: no comparator group; use of treatment other than somatostatin; inappropriate design, such as case reports, animal studies, case series, or gray literature; overlapping or non-extractable data; and outcomes not of interest.

The screening process consisted of two phases: title and abstract screening and full-text screening. In both phases, two reviewers (BS, SA, MA, and RHS) independently assessed each paper using Covidence (covidence.org, Melbourne, VIC, Australia). Discrepancies were resolved by discussion between the senior authors (MSB, MT, ME). Only studies meeting the selection criteria were included.

### 2.3 Outcomes

The main outcomes were TLV, TKV, and eGFR. Secondary outcomes included safety, including rates of cholelithiasis and cholecystitis, as well as diarrhea and abdominal pain. The discontinuation rate was also evaluated.

### 2.4 Quality assessment

Each study was independently evaluated for risk of bias using the Cochrane RoB 2 tool for RCTs (RHS, BS, SA, and MA). The assessment covered five domains: the randomization process, assignment to interventions, missing outcome data, measurement of outcomes, and selection of reported results.^16^

### 2.5 Data extraction and synthesis

After a pilot extraction, the Excel data extraction sheet was refined into its final form. Two reviewers (BS, and SA) independently extracted the data, and a third reviewer (RHS) cross-checked and validated the entries. The sheet comprised three sections: study characteristics; baseline participant variables, including numbers in the control and intervention groups, total sample size, age, renal function, and baseline kidney and liver volumes; and (outcomes of interest.

For continuous outcomes such as TLV, TKV, and eGFR, treatment effects were expressed as percent change from baseline and were calculated using the following formula:

Percentchange=[(meanatfollow−up−meanatbaseline)/meanatbaseline]×100

When standard deviations (SDs) of percent change were not reported, they were back-calculated from the reported standard errors or 95% confidence intervals (CIs) when available. If only baseline and follow-up means and SDs were reported, the SD of change was calculated as follows:

$$\text{SD}\;\text{of}\;\text{change}=\left(\sqrt{\text{SD}}{\text{baseline}}^{2}+\text{SD}\;\text{follow}-{\text{up}}^{2}-2\times r\times \text{SD}\;\text{baseline}\times \text{SD}\;\text{follow}-\text{up}\right)$$

When not reported, the correlation coefficient (*r*) was empirically derived from trials that provided sufficient data to calculate it directly using the following formula:

$$r=\left[\text{SD}\;{\text{baseline}}^{2}+\text{SD}\;\text{follow}-{\text{up}}^{2}-\text{SD}\;\text{change}\right]/2\times \text{SD}\;\text{baseline}\times \text{SD}\;\text{follow}-\text{up}$$

All of the aforementioned methods were derived from the Cochrane Handbook 6.5 (see section 10-5-2).^17^ The calculations for each study are shown in detail in Supplementary Table S3.

Finally, for studies with longer follow-up durations (e.g., 2.5 or 3 years), only data corresponding to approximately 1 year were included in the main analysis to maintain consistency across trials. The time points selected for the analysis are summarized in Supplementary Table S4.

### 2.6 Data analysis

The meta-analysis was performed using RevMan (version 7.2.0; The Cochrane Collaboration, London, UK). For outcomes, we calculated the effect sizes using the mean difference (MD) and odds ratio (OR) with the 95% CI. A p-value of <0.05 was considered statistically significant. A random-effects model was applied in view of the anticipated clinical and methodological heterogeneity across studies, including differences in patient populations, type of somatostatin analog used, and treatment duration. A subgroup analysis was performed based on use of somatostatin agents according to TLV and TKV.

A sensitivity analysis was performed to assess the impact of each study on the overall effect size by removing each study in turn. Heterogeneity was evaluated using the Q-test and I^2^ statistic. Publication bias was examined using Egger's test and visually using funnel plots when the outcome included more than 10 studies. The certainty of the evidence for each outcome was evaluated using the GRADE (Grading of Recommendations Assessment, Development, and Evaluation) approach. GRADE assessment domains and definitions are shown in Supplementary Table S5.

Finally, trial sequential analysis (TSA) was performed for TLV, TKV, and eGFR using TSA software (version 0.9.5.10 Beta; Copenhagen Trial Unit, Copenhagen, Denmark).^18^ For each outcome, we set type I error at 5% (two-sided) and type II error at 20% (80% power). We selected conservative anticipated effect sizes for TSA based on previously reported MDs for outcomes of somatostatin analog therapy in PLD. Specifically, we used −4% for TLV and −2.5% for TKV, reflecting estimates that are slightly conservative when compared with the reported pooled MDs (−6.4% and −3.7%, respectively).^9^ For eGFR, given the modest and non-significant effect (<1%) in previous studies,^19^^,^^20^ we set the anticipated effect at 2%, representing the smallest clinically relevant change that allows TSA computation.

Both the random-effects model and O'Brien–Fleming α-spending boundaries were applied. We calculated the required information size (RIS) and evaluated whether the cumulative Z-curve crossed the monitoring boundaries, indicating either significant benefit or harm, or the futility boundaries, indicating a lack of significance.

## 3 Results

### 3.1 Study selection and overview of the included studies

**Fig. 1:**
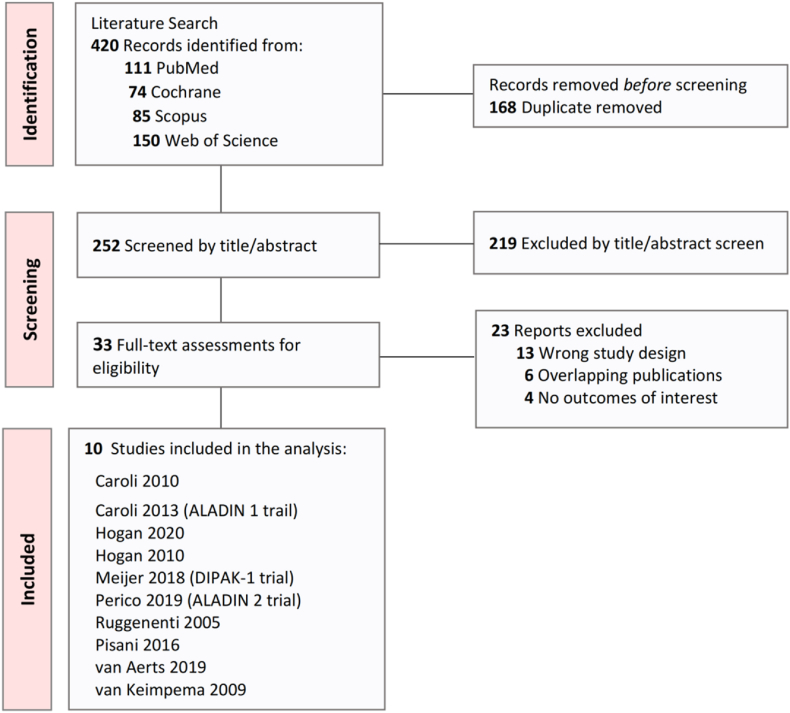
Overview of the article selection process.

The database search yielded 420 potentially relevant articles. Thirty-three studies were reviewed in full text for eligibility, with 10 papers from seven clinical trials ultimately deemed eligible for data extraction and analysis ^13^^,^^14^^,^^20^, ^21^, ^22^, ^23^, ^24^, ^25^, ^26^, ^27^. A detailed illustration of the selection process is presented in Fig. 1.

A total of 640 patients were included in the analysis. Three of the papers were secondary analyses of controlled trials,^14^^,^^23^^,^^26^ six were parallel-group controlled trials,^13^^,^^20^^,^^22^^,^^24^^,^^25^^,^^27^ and one was a crossover trial.^21^ Six trials used a placebo as the control^13^^,^^21^^,^^22^^,^^24^^,^^25^^,^^27^ and one used standard of care.^20^ Four trials used octreotide (40 ​mg),^21^^,^^24^^,^^25^^,^^27^ two used lanreotide (120 ​mg),^20^^,^^22^ and one used pasireotide (60 ​mg).^13^ All trials administered the somatostatin analog as a LAR formulation every 28 days. A detailed overview of the included studies is presented in Table 1. The baseline characteristics of the included patients are summarized in Supplementary Table S6.

**Table 1:** Background overview of the included studies.

Study	Country	Design	Total N	Intervention	Control	Outcomes	Imaging modality	Follow up duration
Ruggenenti 2005^21^	Italy	RCT-cross over	12	Octreotide 40 ​mg IM, every 28 days	Placebo	TKV, eGFR	CT scan	6 months
van Keimpema 2009 (LOCKCYST trial)^22^	Netherlands	RCT-double-blind	54	Lanreotide 120 ​mg IM, every 28 days	Placebo	TLV, TKV	CT scan	6 months
Caroli 2010^23^	Italy	Secondary analysis of Ruggenenti	12	Octreotide 40 ​mg IM, every 28 days	Placebo	TLV	CT scan	6 months
Hogan 2010^24^	US	RCT-double-blind	42	Octreotide 40 ​mg IM, every 28 days	Placebo	TLV, TKV, eGFR	MRI a	1 year
Caroli 2013 (ALADIN trial)^25^	Italy	RCT-Single-blind	79	Octreotide 40 ​mg IM, every 28 days	Placebo	TKV, htTKV, eGFR	MRI	1, and 3 years
Pisani 2016^26^	Italy	Secondary analysis of ALADIN	27	Octreotide 40 ​mg IM, every 28 days	Placebo	htTLV	MRI	3 years
Meijer 2018 (DIPAK-1 trial)^20^	Netherlands	RCT-open label	305	Lanreotide 120 ​mg IM, every 28 days	Standard care only	TKV, htTKV, eGFR	MRI	2.5 years
Perico 2019 (ALADIN-2 trial)^27^	Italy	RCT-double-blind	100	Octreotide 40 ​mg IM, every 28 days	Placebo	TKV, htTKV, eGFR	CT scan	1, and 3 years
Van Aerts 2019^14^	Netherlands	Secondary analysis of DIPAK-1	175	Lanreotide 120 ​mg IM, every 28 days	Standard care only	htTLV	MRI	2.5 years
Hogan 2020^13^	US	RCT-double-blind	48	Pasireotide 60 ​mg IM, every 28 days	Placebo	htTLV, htTKV, eGFR	MRI	1 year

a CT-scan was used in three patients. Abbreviations: RCT, randomised clinical trials; eGFR, estimated glomerular filtration rate; TLV, total liver volume; TKV, total kidney volume; htTLV, hight-adjusted total liver volume; htTKV, hight-adjusted total kidney volume; IM, intramuscular; CT scan, computed tomography scan; MRI magnetic resonance imaging.

### 3.2 Risk of bias assessment

Four studies, namely, those by Ruggenenti (2005),^21^ Caroli (2010),^23^ Perico (2019),^27^ and van Keimpema (2009),^22^ had an overall low risk of bias. Six studies, reported by Hogan (2010),^24^ Hogan (2020),^13^ Caroli (2013),^25^ Pisani (2016),^26^ Meijer (2018),^20^ and van Aerts (2019),^14^ raised some concerns, primarily because of insufficient information on allocation concealment or lack of blinding of physicians and nurses. A detailed summary is presented in Supplementary Fig. S1.

### 3.3 Primary outcomes

#### 3.3.1 Total liver volume

**Fig. 2:**
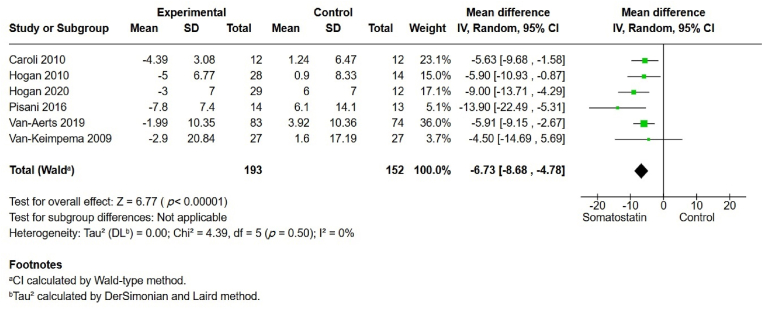
Total liver volume (TLV).

Changes in TLV were reported in six studies (Fig. 2). A significant difference in percent change in TLV was observed between the somatostatin group and the control group, with an MD of −6.73 (95% CI –8.68, −4.78, *p* ​= ​0.001], indicating a significant benefit of somatostatin analogs on TLV. The effect size showed low heterogeneity (I^2^ ​= ​0%). The GRADE assessment was low, which was attributed to the fact that the original trials were designed for ADPKD, with PLD evaluated only as a secondary outcome.

**Fig. 3:**
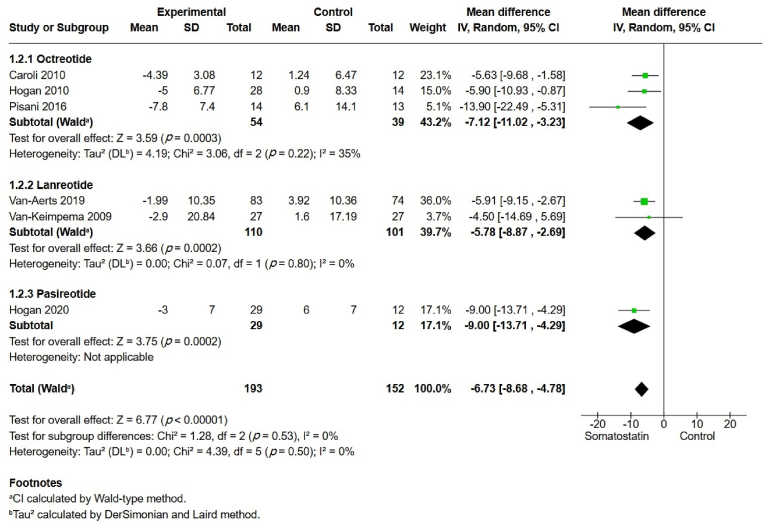
Total liver volume subgroups.

In a subgroup analysis based on type of somatostatin analog (Fig. 3), all three agents (octreotide, lanreotide, and pasireotide) were associated with a significant difference in TLV but without a significant modifying subgroup effect (*p* ​= ​0.53). The small number of included studies may have limited the validity of this subgroup analysis.

#### 3.3.2 Total kidney volume

**Fig. 4:**
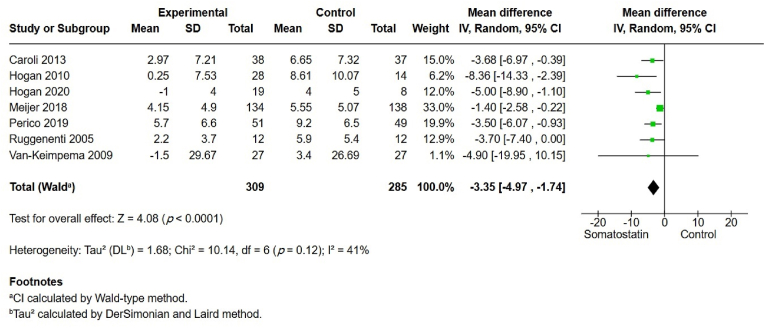
Total kidney volume (TKV).

TKV was reported in seven studies (Fig. 4). There was a significant difference in TKV between the somatostatin group and the control group, with an MD of −3.35 (95% CI –4.97, −1.74, *p* ​< ​0.001). Moderate heterogeneity was observed (I^2^ ​= ​41%). The GRADE assessment was moderate.

A subgroup analysis by type of somatostatin analog revealed significant reductions in TKV across all agents, with a significant subgroup effect (*p* ​= ​0.02; Supplementary Fig. S2). The subgroup analysis was limited by the small number of studies, so this finding should be interpreted with caution.

#### 3.3.3 Estimated glomerular filtration rate

**Fig. 5:**
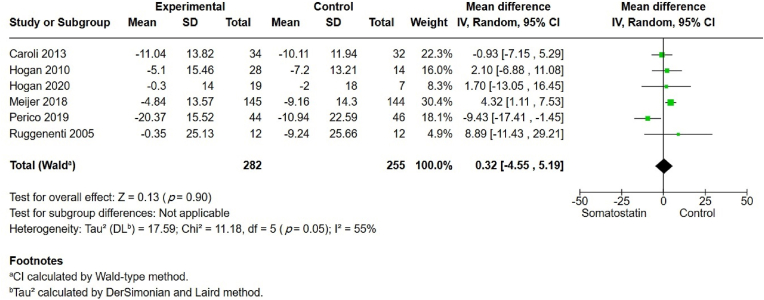
Estimated glomerular filtration rate (eGFR).

The eGFR was reported in six studies, with no significant difference observed between the somatostatin and control groups (MD 0.32, 95% CI –4.55, 5.19, *p* ​= ​0.9) (Fig. 5). Moderate heterogeneity was observed (I^2^ ​= ​55%). GRADE assessment was low as a result of imprecision.

A sensitivity analysis was performed to assess the impact of each study on the overall effect size for TLV, TKV, and eGFR. Each study was removed individually, and no significant changes were observed in the primary analysis.

### 3.4 Safety outcomes

Information on cholelithiasis and cholecystitis was reported in four studies. Treatment with a somatostatin analog was associated with significantly higher adverse event rates (OR 4.66, 95% CI 1.19–18.26, *p* ​= ​0.03; 4 studies; GRADE, moderate]. No significant heterogeneity was observed (I^2^ ​= ​0%). However, heterogeneity assessment was limited by the small sample size and small number of studies (4).

Abdominal pain was significantly more common in the somatostatin group than in the control group (OR 2.82, 95% CI 1.44–5.52, *p* ​= ​0.003; 5 studies; GRADE, moderate], as was diarrhea (OR 85, 95% CI 2.34–10.04, *p* ​< ​0.001; 6 studies; GRADE, moderate). Both outcomes showed a high degree of heterogeneity (I^*2*^ of 66% for abdominal pain and 68% for diarrhea). However, heterogeneity estimates were based on a small number of studies and should therefore be interpreted with caution.

Finally, six studies reported discontinuation rates, with no significant between-group differences noted (OR 2.06, 95% CI 0.55–7.75, *p* ​= ​0.29; 6 studies; GRADE, low). A detailed analysis of the safety outcomes is presented in Supplementary Figs. S3 and S4.

A summary of the overall findings is provided in Table 2. The detailed GRADE assessment for each outcome is summarized in Supplementary Table S5.

**Table 2:** Summary of overall findings.

Outcome	No. of studies	Total No.	Test	Effect	95% CI	*p*-value	I^2¥^	GRADE assessment
Total liver volume	6	345	MD	−6.73	[−8.68, −4.78]	<0.001	0%	⊕⊕⊖⊖ low a
Total Kidney volume	7	594	MD	−3.35	[−4.97, −1.74]	<0.001	41%	⊕⊕⊕⊖ moderate b
eGFR	6	537	MD	0.32	[−4.55, 5.19]	0.90	55%	⊕⊕⊖⊖ low c
Cholelithiasis and cholecystitis	4	496	OR	4.66	[1.19, 18.26]	0.03	0%	⊕⊕⊕⊖ moderate d
Abdominal pain	5	580	OR	2.82	[1.44, 5.52]	0.003	66%	⊕⊕⊕⊖ moderate d
Diarrhea	6	592	OR	4.85	[2.34, 10.04]	<0.001	68%	⊕⊕⊕⊖ moderate d
Discontinuation rate	6	608	OR	2.06	[0.55, 7.75]	0.29	57%	⊕⊕⊖⊖ low c

A *p*-value of <0.05 is statistically significant.

Abbreviations: eGFR, estimated glomerular filtration rate; MD, mean difference; OR, odds ratio; CI: confidence interval.

¥. Heterogeneity.

a Downgraded for indirectness as the original trials were on ADPKD, and PLD was a secondary outcome.

b A proper assessment of imprecision and publication bias was not possible, less than 10 studies.

c Included the null value (downgrade by 2 levels).

d Wide confidence intervals, but the null was not included (downgrade by 1 level).

The results of our sensitivity analyses, conducted by removing studies one at a time, are presented in Supplementary Table S7. For TLV, pooled MDs remained consistently negative across all exclusions, ranging from −6.26 to −7.19. Effect estimates for TKV were similarly stable, ranging from −2.70 to −4.16. For eGFR, pooled estimates remained non-significant, close to the null (range −1.63, 3.16). For safety outcomes, the effect estimates were as follows: cholelithiasis/cholecystitis (OR 3.12–7.54), abdominal pain (OR 3.64–8.84), and diarrhea (OR 5.78–15.16). Overall, no single study materially influenced the direction or magnitude of the pooled effects.

Finally, eight of the included studies used intention-to-treat analyses and three used per-protocol analyses, with attrition rates ranging from 10% to 15%.^13^^,^^14^^,^^20^ Outcomes were pooled as reported, and sensitivity analyses excluding the per-protocol trials were performed to assess the robustness of the findings. The results of the sensitivity analyses were consistent with those of the primary analysis.

### 3.5 Trial sequential analysis

**Fig. 6:**
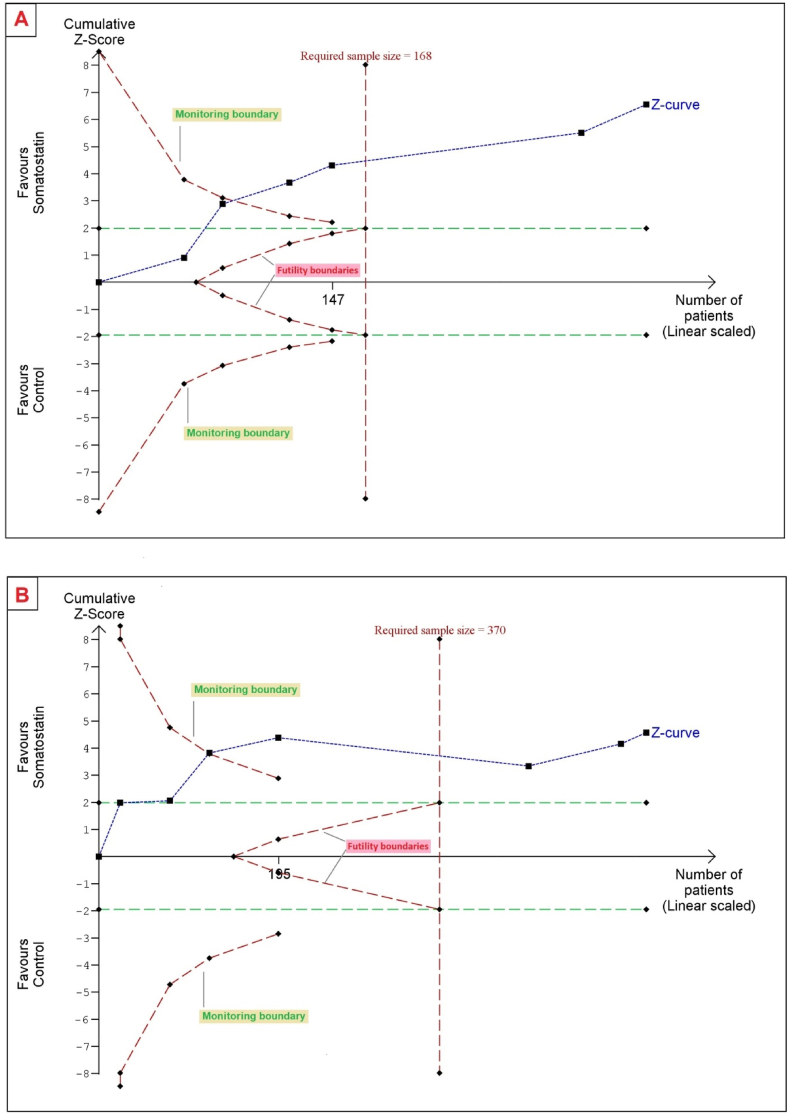
Trial sequential analysis: TLV (A) and TKV (B).

For TLV, the cumulative Z-curve crossed the trial sequential monitoring boundary for benefit and reached the RIS, providing good evidence that somatostatin analogs reduce TLV in comparison with placebo (Fig. 6A).

For TKV, the Z-curve also crossed the benefit boundary and reached the RIS, providing strong evidence of benefit (Fig. 6B).

**Fig. 7:**
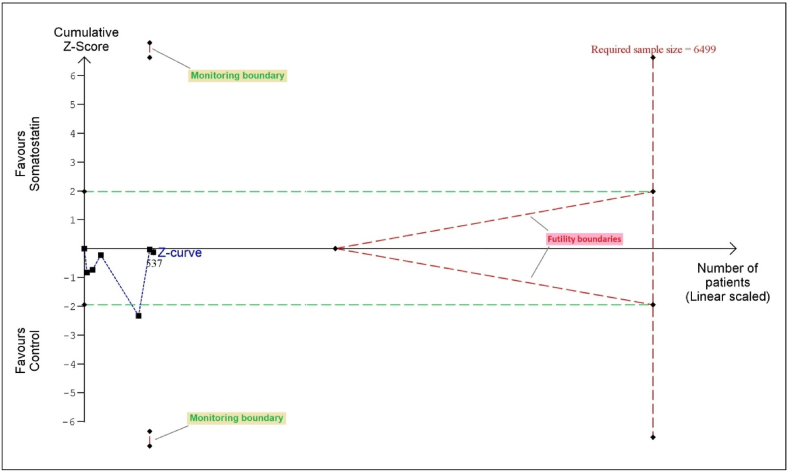
Trial sequential analysis: eGFR.

For eGFR, the Z-curve remained within the conventional boundaries, did not cross the benefit or futility boundaries, and the RIS was not reached, indicating that the evidence for benefit remains inconclusive (Fig. 7).

### 3.6 Assessment of publication bias

This assessment was not possible because of the small number of included studies (<10).

## 4 Discussion

### 4.1 Main findings

This systematic review and meta-analysis evaluated the effects of LAR somatostatin analogs in adults with PLD or ADPKD. Based on low-to-moderate evidence, the percent changes in TLV and TKV were significantly lower in the somatostatin group than in the control group. However, there was no significant between-group difference in percent change in eGFR. A significantly higher incidence of adverse events, including cholelithiasis, cholecystitis, diarrhea, and abdominal pain, was observed in the somatostatin group. However, there was no significant between-group difference in the treatment discontinuation rate.

TSA showed good evidence of benefit for TLV and TKV, with the Z-curves crossing the benefit boundaries and reaching the RIS. For eGFR, the Z-curve stayed within conventional boundaries and did not reach the RIS, leaving the evidence inconclusive.

### 4.2 In the context of current literature

The effects of somatostatin analogs on PLD and ADPKD have been evaluated in previous meta-analyses.^19^^,^^28^^,^^29^ However, the findings regarding their effects on TLV and TKV are conflicting.

One meta-analysis of somatostatin analogs in ADPKD and PLD found a significant reduction in TLV but no significant difference in TKV.^28^ This analysis used post-treatment values, which may have been confounded by baseline variability. The baseline TKV varied widely across the included studies, ranging from 1000 to 2500 ​mL/m.

Another meta-analysis, which used pre-treatment and post-treatment values and calculated the MD, did not find a significant difference in TLV between the somatostatin group and the control group.^29^ However, the included studies reported significant differences in TLV. Use of absolute values in the presence of baseline heterogeneity may have influenced the results. In contrast, our meta-analysis used percent change from baseline and demonstrated a significant reduction in both TLV and TKV. Our results are concordant with those observed in clinical trials ^14^^,^^24^, ^25^, ^26^.

A further meta-analysis of studies on somatostatin analogs in ADPKD and PLD was conducted in 2021.^19^ As in our study, the investigators used the percent change from baseline. However, some methodological differences were noted. All of our calculations are based on the Cochrane Handbook (version 6.5, section 10.5.2).^17^ Hence, the slight differences in effect sizes’ numerical values. Moreover, we performed subgroup analyses for TLV and TKV by specific somatostatin analog, in view of the differences in activity found in animal and human studies.^12^^,^^30^ Furthermore, we conducted TSA for TLV, TKV, and eGFR.

### 4.3 Implications for clinical care and research

While reductions in liver and kidney volume are important surrogate markers, their clinical relevance depends on whether they translate into improvements in symptoms and quality of life for patients. A previous study found that liver size is a significant predictor of quality of life in patients with PLD.^31^

The most common symptoms associated with PLD are abdominal discomfort and pain, and the symptom burden can significantly impact quality of life.^32^ The primary goal of treatment is to reduce the size of cysts, thereby relieving symptoms and improving quality of life, as well as reducing the impact of cyst volume on liver and kidney function.

Another study also demonstrated that somatostatin therapy improved health-related quality of life in patients with PLD by reducing cyst size.^32^ It is worth mentioning that the existing data also suggest that the benefits of LAR somatostatin analogs persist after discontinuation.^26^ Given that PLD is an indication for liver transplantation in individuals with progressive disease and has an impact on hepatic synthetic function, therapies that can improve TLV are of significant interest, given that they may avoid end-stage liver disease.

Somatostatin may have long-term benefits in patients with PLD, with some patients maintaining a 10% reduction in TLV even after treatment cessation, as demonstrated by Pisani et al.^26^ The potential of an intermittent (“on-and-off”) treatment strategy to sustain these benefits and reduce costs warrants further investigation.

Given the high rates of co-existing polycystic kidney disease (PKD) among individuals with PLD, a similar benefit of LAR somatostatin analogs in terms of reducing TKV would be noteworthy. Interestingly, although TKV was reduced, there was no significant change in eGFR in this meta-analysis, suggesting that the impact on renal function in PKD is likely mediated by factors beyond kidney cyst volume alone. In the ALADIN 2 trial,^27^ progression to end-stage renal disease or doubling of serum creatinine was slower in patients with stage 4 chronic kidney disease who received a somatostatin analog than in those who received placebo despite no significant change in eGFR. Furthermore, TSA indicated that the available evidence for eGFR was inconclusive, and further adequately powered RCTs are needed to clarify the effect of somatostatins on renal function in ADPKD.

Tolvaptan, a vasopressin V2 receptor antagonist, is the only agent that has demonstrated improvements in both TKV and eGFR in ADPKD, as shown in the 3-year TEMPO trial.^33^ However, its use is limited by a high discontinuation rate because of adverse effects, such as thirst, nocturia, polyuria, and polydipsia, as well as high cost. A recent pilot trial demonstrated a significant improvement in eGFR in patients who received a combination of tolvaptan and LAR octreotide in comparison with those who received tolvaptan plus placebo.^34^ These findings highlight the potential of combining agents with complementary mechanisms as a way of overcoming the limitations of the therapies available for PLD and PKD. Large RCTs are needed to confirm these findings and to establish the safety and tolerability of these combinations.

While no significant subgroup differences were observed for TLV, significant differences were noted for TKV. This finding may reflect differences in expression of somatostatin receptor subtypes in renal tissue and variations in receptor affinity among individual agents.^10^ However, this finding should be considered exploratory. The very small number of trials within each subgroup renders formal tests underpowered and vulnerable to false-positive results. Accordingly, the observed differences are insufficient to support conclusions regarding differential efficacy among individual somatostatin analogs.

Our study found a significantly higher incidence of cholecystitis and biliary stones in somatostatin group compared to control, which are known to be common with this class of medications.^35^ Elevated blood glucose levels have also been reported, necessitating careful monitoring in patients with diabetes.^13^^,^^20^ Furthermore, LAR somatostatin analogs are associated with substantial financial costs, which may limit their accessibility and use.^36^

The increased rates of abdominal pain observed in this meta-analysis may be partly explained by the overlap in the rates of abdominal pain and cholelithiasis/cholecystitis reported in several studies^20^^,^^25^^,^^27^ and underscore the importance of careful monitoring and patient counseling when initiating therapy.

We found no significant between-group difference in the treatment discontinuation rate. However, some studies have reported dose reductions or withholding in the somatostatin arm. Therefore, relying solely on discontinuation rates to assess treatment tolerability may be misleading. In our study, we used a random-effects model, whereas another meta-analysis that used a fixed-effects model found a significantly higher discontinuation rate in the somatostatin group.^28^ We believe that using a random-effects model provides a more cautious and generalizable estimate.

The ongoing AGAINST-PLD study is an RCT comparing leuprorelin, a gonadotropin-releasing hormone agonist, with standard of care in premenopausal women with PLD.^37^ The rationale for the study is that targeting estrogen pathways could offer therapeutic benefit because PLD is more common in younger women of reproductive age and estrogen may promote liver growth.^38^^,^^39^

Multicenter RCTs are needed to identify which patients with PLD would benefit most from LAR somatostatin therapy. More meaningful insights could be obtained by evaluating its effects across different age groups and disease stages using standardized selection criteria. Future research could draw on successful experiences in other fields, such as using proteomics and Mendelian randomization methods to identify therapeutic targets in liver cancer.^40^

Additional research is also warranted to assess the potential of combinations of somatostatin analogs and other therapies to delay the progression of liver and kidney disease.

### 4.4 Strengths and limitations

Unlike the previous meta-analyses, a key strength of this study was the method used to assess the impact of somatostatin analog therapy on TLV and TKV. At baseline, the included studies showed significant heterogeneity in total kidney and liver volumes. We used percent change from baseline rather than post-treatment values or absolute changes to address this heterogeneity. Post-treatment values are sensitive to differences at baseline, which can confound treatment effect estimates, especially when baseline sizes are unevenly distributed across studies. Using absolute change from baseline would adjust for some baseline differences but remain sensitive to initial size (Cochrane Handbook 6.5, section 10-5-2).^17^ However, percentage change standardizes outcomes relative to baseline, allowing for more valid comparisons across studies with varying baseline volumes. Percentage change reduces the influence of initial size differences and better reflects the relative treatment effect. Furthermore, some of the included studies reported height-adjusted TKV, while others used absolute TKV; thus, percent change provides a more consistent metric for comparing these differences.

This meta-analysis also has some limitations. The number of included studies was small, and most had relatively small sample sizes. Moreover, most of the studies were performed in the USA, Italy, or the Netherlands, which may limit the generalizability of the findings to other ethnic groups. In some of the included trials, liver outcomes were not part of the original study design. In others, they were secondary endpoints, with most being focused primarily on kidney function and eGFR in ADPKD, making it difficult to extrapolate efficacy to patients with PLD alone. Few patients in these studies had isolated PLD, likely because of the rarity of this disease and the associated recruitment challenges.

Most of the included studies had follow-up durations of less than 5 years, which limited our ability to assess long-term efficacy and safety. Studies with longer follow-up durations are needed to determine the durability of the reduction in TLV and to better characterize the long-term safety profile of somatostatin analogs.

The small number of studies also limited our ability to perform subgroup analyses based on type of somatostatin agent. There was also variability in treatment duration across studies, which could potentially influence percent change outcomes. It was impossible to assess the effect at different stages of disease progression. Furthermore, the impact of somatostatin on quality of life was evaluated in only a few trials. Five of the included studies had concerns in their risk of bias assessment. Given the small number of studies for each outcome, estimates of between-study heterogeneity, such as the I^2^ statistic, may have been imprecise and not reflect true variability across studies.^41^ Finally, we could not assess publication bias because of the small number of studies (<10).

## 5 Conclusions

Based on low-to-moderate evidence, LAR somatostatin analogs achieved a reduction in the progression of both TLV and TKV in comparison with controls, as evidenced by a lower percent change from baseline in the somatostatin arm. However, there was no significant between-group difference in the decline in eGFR. Furthermore, a higher incidence of adverse events, including cholelithiasis, cholecystitis, diarrhea, and abdominal pain, was observed in the somatostatin group.

Larger controlled trials are needed to evaluate further the ability of somatostatin analogs to delay progression or PLD and PKD, establish their long-term use in these diseases, and determine their safety profile. There is still a need for safe and tolerable therapies that can delay the progressive deterioration of liver and kidney function seen in patients with PLD and PKD.

## Animal treatment

Not applicable.

## CRediT authorship contribution statement

**Mohammed S. Beshr:** Writing – review & editing, Writing – original draft, Visualization, Software, Methodology, Investigation, Conceptualization. **Rana H. Shembesh:** Writing – review & editing, Writing – original draft, Methodology, Investigation, Conceptualization. **Bisher Sawaf:** Writing – review & editing, Methodology, Investigation, Data curation. **Shahem Abbarh:** Writing – review & editing, Methodology, Investigation, Data curation. **Mohammed Abu-Rumaileh:** Writing – review & editing, Methodology, Investigation, Data curation. **Monica Tincopa:** Writing – review & editing, Supervision, Methodology, Investigation, Data curation. **Muhammed Elhadi:** Writing – review & editing, Writing – original draft, Supervision, Project administration, Methodology, Investigation, Formal analysis, Data curation, Conceptualization.

## Informed consent

Not applicable. This study was solely based on previously published articles.

## Organ donation

Not applicable.

## Ethics statement

Not applicable.

## Data availability statement

Data is available upon reasonable request from the corresponding author.

## Declaration of generative AI and AI-assisted technologies in the writing process

During manuscript preparation, the authors used ChatGPT-4 exclusively for language and grammar refinement. The tool was not used for study design, methodology, or data analysis. All content was subsequently reviewed and edited by the authors, who take full responsibility for the final published work.

## Funding

No financial funding was provided to this project.

## Declaration of competing interest

The authors have nothing to declare.
