# Supplementary material for: Somatostatin analog therapy in delaying progression of polycystic liver disease: A meta-analysis with trial sequential analysis
Source: ILIVER. 2026 Jan 16;5(1):100220. doi: 10.1016/j.iliver.2026.100220 (PMC12870861; doi:10.1016/j.iliver.2026.100220)
Supplement: Multimedia component 1 [file mmc1.docx]

**Supplementary Online Content**

This supplementary material has been provided by the authors for the following paper:

*Effectiveness of Somatostatin Analog Therapy in Delaying Progression of Polycystic Liver Disease: A Meta-Analysis with Trial Sequential Analysis*

**Contents**

[Supplementary Table S1 Prisma 2020 checklist 2](#_Toc219316801)

[Supplementary Table S2. Database search results 6](#_Toc219316802)

[Supplementary Table S3. Calculations of Percent Change from Baseline Mean and Standard Deviation 7](#_Toc219316803)

[Supplementary Table S4. Time Points for Data Extraction of Key Outcomes 11](#_Toc219316804)

[Supplementary Table S5. Summary of GRADE assessment of the reported outcomes 12](#_Toc219316805)

[Supplementary Table S6. Baseline characteristics of the included patients 13](#_Toc219316806)

[Supplementary Table S7. Leave-one-out sensitivity analyses for TLV, TKV, and eGFR 14](#_Toc219316807)

[Supplementary Figure S1. Risk of bias assessment 15](#_Toc219316808)

[Supplementary Figure S2. Total kidney volume, subgroup analysis 16](#_Toc219316809)

[Supplementary Figure S3. Safety outcomes analysis 17](#_Toc219316810)

[Supplementary Figure S4. Discontinuation rate 18](#_Toc219316811)

| **Section and Topic** | **Item #** | **Prisma 2020 Checklist item for abstract** | **Reported (Yes/No)** |
| --- | --- | --- | --- |
| **TITLE** | | |  |
| Title | 1 | Identify the report as a systematic review. | Yes |
| **BACKGROUND** | | |  |
| Objectives | 2 | Provide an explicit statement of the main objective(s) or question(s) the review addresses. | Yes |
| **METHODS** | | |  |
| Eligibility criteria | 3 | Specify the inclusion and exclusion criteria for the review. | Yes |
| Information sources | 4 | Specify the information sources (e.g. databases, registers) used to identify studies and the date when each was last searched. | Yes |
| Risk of bias | 5 | Specify the methods used to assess risk of bias in the included studies. | No |
| Synthesis of results | 6 | Specify the methods used to present and synthesise results. | Yes |
| **RESULTS** | | |  |
| Included studies | 7 | Give the total number of included studies and participants and summarise relevant characteristics of studies. | Yes |
| Synthesis of results | 8 | Present results for main outcomes, preferably indicating the number of included studies and participants for each. If meta-analysis was done, report the summary estimate and confidence/credible interval. If comparing groups, indicate the direction of the effect (i.e. which group is favoured). | Yes |
| **DISCUSSION** | | |  |
| Limitations of evidence | 9 | Provide a brief summary of the limitations of the evidence included in the review (e.g. study risk of bias, inconsistency and imprecision). | No |
| Interpretation | 10 | Provide a general interpretation of the results and important implications. | Yes |
| **OTHER** | | |  |
| Funding | 11 | Specify the primary source of funding for the review. | No |
| Registration | 12 | Provide the register name and registration number. | Yes |

**Supplementary Table S1 Prisma 2020 checklist**

| **Section/Topic** | **Item #** | **Prisma 2020 Checklist item for manuscript** | **Location** |
| --- | --- | --- | --- |
| **TITLE** | | |  |
| Title | 1 | Identify the report as a systematic review. | 1 |
| **ABSTRACT** | | |  |
| Abstract | 2 | See the PRISMA 2020 for Abstracts checklist. | Checked |
| **INTRODUCTION** | | |  |
| Rationale | 3 | Describe the rationale for the review in the context of existing knowledge. | 5 |
| Objectives | 4 | Provide an explicit statement of the objective(s) or question(s) the review addresses. | 6 |
| **METHODS** | | |  |
| Eligibility criteria | 5 | Specify the inclusion and exclusion criteria for the review and how studies were grouped for the syntheses. | 6 |
| Information sources | 6 | Specify all databases, registers, websites, organisations, reference lists and other sources searched or consulted to identify studies. Specify the date when each source was last searched or consulted. | 6 |
| Search strategy | 7 | Present the full search strategies for all databases, registers and websites, including any filters and limits used. | Suppl table S1 |
| Selection process | 8 | Specify the methods used to decide whether a study met the inclusion criteria of the review, including how many reviewers screened each record and each report retrieved, whether they worked independently, and if applicable, details of automation tools used in the process. | 6 |
| Data collection process | 9 | Specify the methods used to collect data from reports, including how many reviewers collected data from each report, whether they worked independently, any processes for obtaining or confirming data from study investigators, and if applicable, details of automation tools used in the process. | 7 |
| Data items | 10a | List and define all outcomes for which data were sought. Specify whether all results that were compatible with each outcome domain in each study were sought (e.g. for all measures, time points, analyses), and if not, the methods used to decide which results to collect. | 7 |
|  | 10b | List and define all other variables for which data were sought (e.g. participant and intervention characteristics, funding sources). Describe any assumptions made about any missing or unclear information. |  |
| Study risk of bias assessment | 11 | Specify the methods used to assess risk of bias in the included studies, including details of the tool(s) used, how many reviewers assessed each study and whether they worked independently, and if applicable, details of automation tools used in the process. | 7 |
| Effect measures | 12 | Specify for each outcome the effect measure(s) (e.g. risk ratio, mean difference) used in the synthesis or presentation of results. | 8 |
| Synthesis methods | 13a | Describe the processes used to decide which studies were eligible for each synthesis (e.g. tabulating the study intervention characteristics and comparing against the planned groups for each synthesis (item #5)). | N/A |
|  | 13b | Describe any methods required to prepare the data for presentation or synthesis, such as handling of missing summary statistics, or data conversions. | 7 |
|  | 13c | Describe any methods used to tabulate or visually display results of individual studies and syntheses. | N/A |
|  | 13d | Describe any methods used to synthesize results and provide a rationale for the choice(s). If meta-analysis was performed, describe the model(s), method(s) to identify the presence and extent of statistical heterogeneity, and software package(s) used. | 7 |
|  | 13e | Describe any methods used to explore possible causes of heterogeneity among study results (e.g. subgroup analysis, meta-regression). | 8 |
|  | 13f | Describe any sensitivity analyses conducted to assess robustness of the synthesized results. | 8 |
| Reporting bias assessment | 14 | Describe any methods used to assess risk of bias due to missing results in a synthesis (arising from reporting biases). | 8 |
| Assessment | 15 | Describe any Certainty methods used to assess certainty (or confidence) in the body of evidence for an outcome. | 8 |
| **RESULTS** | | |  |
| Study selection | 16a | Describe the results of the search and selection process, from the number of records identified in the search to the number of studies included in the review, ideally using a flow diagram. | 8 and Fig 1 |
|  | 16b | Cite studies that might appear to meet the inclusion criteria, but which were excluded, and explain why they were excluded. | NA |
| Study characteristics | 17 | Cite each included study and present its characteristics. | Suppl Table S2 |
| Risk of bias in studies | 18 | Present assessments of risk of bias for each included study. | 9 |
| Results of individual studies | 19 | For all outcomes, present, for each study: (a) summary statistics for each group (where appropriate) and (b) an effect estimate and its precision (e.g. confidence/credible interval), ideally using structured tables or plots. | 9-10,  Fig 2-6, Suppl fig S3 |
| Results of syntheses | 20a | For each synthesis, briefly summarise the characteristics and risk of bias among contributing studies. | Suppl fig 1 |
|  | 20b | Present results of all statistical syntheses conducted. If meta-analysis was done, present for each the summary estimate and its precision (e.g. confidence/credible interval) and measures of statistical heterogeneity. If comparing groups, describe the direction of the effect. | 9-10 |
|  | 20c | Present results of all investigations of possible causes of heterogeneity among study results. | 9-10 |
|  | 20d | Present results of all sensitivity analyses conducted to assess the robustness of the synthesized results. | 9-10 |
| Reporting biases | 21 | Present assessments of risk of bias due to missing results (arising from reporting biases) for each synthesis assessed. | 10 and Suppl fig S2A and S2B |
| Certainty of evidence | 22 | Present assessments of certainty (or confidence) in the body of evidence for each outcome assessed. | 8-9 supp Table 3 |
| **DISCUSSION** | | |  |
| Discussion | 23a | Provide a general interpretation of the results in the context of other evidence. | 11 |
|  | 23b | Discuss any limitations of the evidence included in the review. | 13 |
|  | 23c | Discuss any limitations of the review processes used. | 13 |
|  | 23d | Discuss implications of the results for practice, policy, and future research. | 13 |
| **OTHER INFORMATION** | | |  |
| Registration and protocol | 24a | Provide registration information for the review, including register name and registration number, or state that the review was not registered. | 6 |
|  | 24b | Indicate where the review protocol can be accessed, or state that a protocol was not prepared. | 6 |
|  | 24c | Describe and explain any amendments to information provided at registration or in the protocol. | N/A |
| Support | 25 | Describe sources of financial or non-financial support for the review, and the role of the funders or sponsors in the review. | 2 |
| Competing interests | 26 | Declare any competing interests of review authors. | 2 |
| Availability of data, code and other materials | 27 | Report which of the following are publicly available and where they can be found: template data collection forms; data extracted from included studies; data used for all analyses; analytic code; any other materials used in the review. | 2 |

Supplementary Table S2. Database search results

| **Data Base** | **Search Query** | **N** |
| --- | --- | --- |
| **PubMed**  No filters applied,  Date: 05/1/2025 | ("octreotide" OR "Octreotide" OR "somatostatin analogues" OR "Sandostatin" OR "Octreotide Acetate" OR "Sandostatine" OR "Octreotide"[Mesh] OR lanreotide OR pasireotide OR SOM230 OR "SOM-230") AND ("Isolated autosomal dominant polycystic liver disease" OR "Isolated polycystic liver disease" OR "polycystic liver disease" OR "autosomal dominant polycystic liver disease" OR ADPLD OR "autosomal dominant polycystic kidney disease" OR ADPKD) | 111 |
| **Scopus**  TITLE-ABS-KEY Articles only filter  Date: 05/1/2025 | ("Octreotide" OR "somatostatin analogue*" OR "Sandostatin" OR "Octreotide Acetate" OR "Sandostatine" OR lanreotide OR pasireotide OR SOM230 OR "SOM-230") AND ("Isolated autosomal dominant polycystic liver disease" OR "Isolated polycystic liver disease" OR "polycystic liver disease" OR "autosomal dominant polycystic liver disease" OR ADPLD OR "autosomal dominant polycystic kidney disease" OR ADPKD) | 85 |
| **Cochrane** Filter: trials  Title Abstract Keyword  Date: 05/1/2025 | ("Octreotide" OR "somatostatin analogue*" OR "Sandostatin" OR "Octreotide Acetate" OR "Sandostatine" OR lanreotide OR pasireotide OR SOM230 OR "SOM-230") AND ("Isolated autosomal dominant polycystic liver disease" OR "Isolated polycystic liver disease" OR "polycystic liver disease" OR "autosomal dominant polycystic liver disease" OR ADPLD OR "autosomal dominant polycystic kidney disease" OR ADPKD) | 74 |
| **Web of Science**  No filter Date: 05/1/2025 | ("Octreotide" OR "somatostatin analogue*" OR "Sandostatin" OR "Octreotide Acetate" OR "Sandostatine" OR lanreotide OR pasireotide OR SOM230 OR "SOM-230") AND ("Isolated autosomal dominant polycystic liver disease" OR "Isolated polycystic liver disease" OR "polycystic liver disease" OR "autosomal dominant polycystic liver disease" OR ADPLD OR "autosomal dominant polycystic kidney disease" OR ADPKD) | 150 |

Supplementary Table S3. Calculations of Percent Change from Baseline Mean and Standard Deviation

| **Van Keimpema 2009 [22], TLV**  SST percent change Mean (95% CI) = −2.9% (−11.1% to 5.4%), *n* = 27 Control percent change Mean (95% CI) = 1.6% (−5.2% to 8.4%), *n* = 27  We calculated the SD. Because of the small sample size, we used the t-distribution; for a 95% confidence level with *n* = 27 (degrees of freedom = 26), the critical t-value is approximately 2.056. (Cochrane handbook Version 6.5, 2024, 6.5.2.2)  SD = [(Upper CI−Lower CI)/2 × t] × √*n*  SST percent change Mean (SD) = −2.9 (20.84) Control percent change Mean (SD) = 1.6 (17.19) |
| --- |
| **Van Keimpema 2009 [22], TKV** SST percent change Mean (95% CI) = −1.5% (−13.2% to 10.3%), *n* = 27  Control percent change Mean (95% CI) = 3.4% (−7.1% to 14.0%), *n* = 27  SD = [(Upper CI−Lower CI)/2 × t] × √*n*, *t*-value is approximately 2.056  SST percent change Mean (SD) = −1.5 (29.67) Control percent change Mean (SD) = 3.4 (26.69) |
| **Caroli 2010 [23], TLV** Using the individual patient data in Table 2 sample size was 12. We calculated percent change Mean and SD.  SST percent change Mean (SD) = −4.39% (3.08) Control percent change Mean (SD) = 1.24% (6.47) |
| **Caroli 2013 [25], ALADIN trial TKV at 1-year** SST baseline Mean (SE) = 1556.9 (167.9), *n* = 38 SST after 1-year Mean (SE) = 1603.1 (176.1), *n* = 38 SST absolute 1-year change Mean (SE) = 46.2 (18.2), *n* = 38  Control baseline Mean (SE) = 2161.2 (209.6), *n* = 37 Control after 1-year Mean (SE) = 2304.9 (224.6), *n* = 37 Control absolute 1-year change Mean (SE) = 143.7 (26.0), *n* = 37  1. SD = SE × √*n*  SST baseline Mean (SD) = 1556.9 (1034.3) SST after 1-year Mean (SD) = 1603.1 (1084.8) SST absolute change Mean (SD) = 46.2 (112.18)  Control baseline Mean (SD) = 2161.2 (1274.4) Control after 1-year Mean (SD) = 2304.9 (1365.6) Control absolute 1-year change Mean (SD) = 143.7 (158.16)  2. Correlation r  r = [SD^2^ before + SD^2^ after – SD change^2^]/2 × SD before × SD after  (Cochrane Handbook Version 6.5, 2024, 6.5.2.8)  Correlation r for TKV in SST = 0.9968 Correlation r for TKV in Control = 0.9944  3. Percent change Mean and SD  Percent change mean = [1 year − baseline mean/baseline mean] × 100  Percent Change SD = (SD change/Mean before) × 100 SD change​ = √ [SD^2^ before + SD^2^ after − 2 × r × SD before × SD after]​​  (Cochrane Handbook Version 6.5, 2024, 6.5.2.8)  SST Percent Change Mean (SD) ≈ 2.97 (7.21) Control Percent Change Mean (SD) ≈ 6.65 (7.32) |
| **Caroli 2013 [25], ALADIN trial eGFR at 1 year**  Using WebPlotDigitizer:  SST percent change Mean (SE) = −11.04 (2.37), *n* = 34 Control percent change Mean (SE) = −10.11 (2.11), *n* = 32  SD = SE × √*n*  SST Percent Change Mean (SD) = −11.04 (13.82) Control Percent Change Mean (SD) = −10.11 (11.94) |
| **Meijer 2018 [20], DIPAK-1 trial TKV change 1 year**  SST percent change Mean (95% CI) = 4.15 (3.33 to 4.99), *n* = 134  Control percent change Mean (95% CI) = 5.55 (4.67 to 6.36), *n* = 138  SE = (Upper limit−Lower limit)/2 × 1.96 (Cochrane handbook Version 6.5, 2024, 6.5.2.2)  SD = SE × √*n*  SST percent change Mean (SD) = 4.15 (4.90) Control percent change Mean (SD) = 5.55 (5.07) |
| **Meijer 2018 [20], DIPAK-1 trial eGFR change at 48 weeks**  SST baseline eGFRc Mean (SD) = 51.0 (11.5) Control baseline eGFRc Mean (SD) = 51.4 (11.2)  1. Using WebPlotDigitizer  SST at 48 weeks Mean (95% CI) = 48.53 (46.56 to 50.54), *n* = 145 Control at 48 weeks Mean (95% CI) = 46.69 (44.64 to 48.7), *n* = 144  SE = (Upper limit−Lower limit)/2 × 1.96 SD = SE/√n  SST at 48 weeks Mean (SD) = 48.53 (12.23) Control at 48 weeks Mean (SD) = 46.69 (12.43)  2. calculating r  We calculated r (correlation value), form other studies.  r = [SD^2^ before + SD^2^ after – SD change^2^]/2 × SD before × SD after  Hogan 2010: For STT, SD baseline = 26.53, SD after = 25.66, SD change = 15.46, r = 0.825  For control, SD baseline = 28.08, SD after = 26.40, SD change = 13.21, r = 0.883  Hogan 2020: For STT, SD baseline = 24, SD after = 22, SD change = 14, r = 0.818  For control, SD baseline = 18, SD after = 22, SD change = 18, r = 0.611  Caroli 2013: For STT Baseline SE= 88.68 (3.93), *n* = 36; After SE = 4.23, *n* = 34  For control baseline SE = 5.30, *n* = 34; After SE: 5.45, *n* = 32  For STT, SD baseline = 23.58, SD after = 24.66, SD change = 13.82, r = 0.839  For control, SD baseline = 30.91, SD after = 30.68, SD change = 11.94, r = 0.924  Average r (SST) = (0.825 + 0.818 + 0.839)/3 = 0.827 Average r (Control) = (0.883 + 0.611 + 0.924)/3 = 0.806  3. Percent change Mean = [End – baseline]/baseline × 100  STT percent change mean = −4.84 Control percent change mean = −9.16  4. Percent change SD  Percent Change SD = (SD change/Mean before) × 100 SD change​ = √ [SD^2^ before + SD^2^ after − 2 × r × SD before × SD after]​​  STT percent change Mean (SD) = −4.84 (13.57) Control percent change Mean (SD) = −9.16 (14.30) |
| **Van Aerts 2019 [14], DIPAK-1 trial TLV change**  SST percent change Mean (95% CI) = −1.99 (-4.21 to 0.24), *n* = 83 Control percent change Mean (95% CI) = 3.92 (1.56 to 6.28), *n* = 74  SE = (Upper limit−Lower limit)/2 × 1.96  SD = SE × √n  SST percent change Mean (SD) = −1.99 (10.35) Control percent change Mean (SD) = 3.92 (10.36) |
| **Perico 2019 [27], ALADIN-2 trial, TKV 1 year**  SST Percent change Median (IQR): 5.2 (1.6−10.2), *n* = 51 Control Percent change Median (IQR): 8.8 (5.2−13.7), *n* = 49  Converted to mean and SD using method by Wan et. al. (PMID: 25524443)  SST percent change Mean (SD) = 5.7 (6.6) Control percent change Mean (SD) = 9.2 (6.5) |
| **Perico 2019 [27], ALADIN-2 trial, eGFR 1 year**  SST baseline eGFR Median (IQR) = 31.5 (25.6 to 36.6), *n* = 50 SST End eGFR Median (IQR) = 25.3 (19.4 to 29.9), *n* = 44  Control baseline eGFR Median (IQR) = 30.9 (21.6 to 37.4), *n* = 47 Control End eGFR Median (IQR) = 24.4 (20.9 to 34.8), *n* = 46  1. Converted to mean and SD using method by Wan et. al. (PMID: 25524443)  SST baseline eGFR Mean (SD) = 31.23 (8.4)  SST End eGFR Mean (SD) = 24.87 (8.05)  Control baseline eGFR Mean (SD) = 29.97 (12.08) Control End eGFR Mean (SD) = 26.7 (10.64)  2. Percent change = [End – baseline]/baseline × 100  STT percent change Mean = −20.37 Control percent change Mean = −10.94  Correlation r (SST) = 0.827 Correlation r (Control) = 0.806  3. percent change SD  Percent Change SD = (SD change/Mean before) × 100 SD change​ = √ [SD^2^ before + SD^2^ after − 2 × r × SD before × SD after]​​  SST Percent Change Mean (SD) = −20.37 (15.52) Control Percent Change Mean (SD) = −10.94 (22.59) |
| **Ruggenenti 2005 [21] eGFR**  SST baseline Mean (SD) = 57.9 (22.4) SST after 6 months Mean (SD) = 57.7 (25.7), *n* = 12  Control baseline = 59.5 (25.2) Control after 6 months = 54.0 (23.6), *n* = 12  Correlation r (SST) = 0.827 Correlation r (Control) = 0.806  1. Percent Change  Percent Change = [(mean after – mean baseline)/mean baseline] × 100  SST Percent Change mean = −0.35  Control Percent Change mean = −9.24  3. percent change SD  Percent Change SD = (SD change/Mean before) × 100  SD change = √ [SD^2^ before + SD^2^ after − 2 × r × SD before × SD after]​​  SST Percent Change Mean (SD) = −0.35 (25.13) Control Percent Change Mean (SD) = −9.24 (25.66) |
| Abbreviations: eGFR, estimated glomerular filtration rate; TLV, total liver volume; TKV, total kidney volume; SST, somatostatin; SE, standard error; SD, standard deviation; CI, confidence interval |

Supplementary Table S4. Time Points for Data Extraction of Key Outcomes

| **Study** | **TKV** | **eGFR** | **TLV** |
| --- | --- | --- | --- |
| Caroli 2010 [23] | – | – | 6 months |
| Caroli 2013 [25] | 1 year | 1 year | – |
| Hogan 2010 [24] | 1 year | 1 year | 1 year |
| Hogan 2020 [13] | 1 year | 1 year | 1 year |
| Meijer 2018 [20] | 1 year | 48 weeks | – |
| Perico 2019 [27] | 1 year | 1 year | – |
| Pisani 2016 [26] | – | – | 3 years |
| Ruggenenti 2005 [21] | 6 months | 6 months | – |
| Van-Aerts 2019 [14] | – | – | 1 year |
| Van-Keimpema 2009 [22] | 6 months | – | 6 months |
| Abbreviations: TLV, total liver volume; TKV, total kidney volume; eGFR, estimated glomerular filtration rate. | | | |

Supplementary Table S5. Summary of GRADE assessment of the reported outcomes

| **Outcome** | **Risk of bias** | **Inconsistency** | **Indirectness** | **Imprecision** | **Publication bias** | **Overall rating** |
| --- | --- | --- | --- | --- | --- | --- |
| **TLV** | No concerns | Not applicable | original trials’ design for ADPKD; PLD secondary outcome; downgrade level 2 | No concerns | Not applicable | ⊕⊕⊖⊖  low |
| **TKV** | In one study | Not applicable | Original design for ADPKD; no concerns | No concerns | Not applicable | ⊕⊕⊕⊖  moderate |
| **eGFR** | No concerns | Not applicable | Original design for ADPKD; no concerns | Included the null value | Not applicable | ⊕⊕⊖⊖  low |
| **Cholelithiasis/ cholecystitis** | No concerns | Not applicable | No concerns | Wide CIs | Not applicable | ⊕⊕⊕⊖  moderate |
| **Abdominal pain** | No concerns | Not applicable | No concerns | Wide CIs | Not applicable | ⊕⊕⊕⊖  moderate |
| **Diarrhea** | No concerns | Not applicable | No concerns | Wide CIs | Not applicable | ⊕⊕⊕⊖  moderate |
| **Discontinuation** | No concerns | Not applicable | No concerns | Included the null value | Not applicable | ⊕⊕⊖⊖  low |
| Abbreviations: TLV, total liver volume; TKV, total kidney volume; eGFR, estimated glomerular filtration rate.  The quality of evidence was graded as high, moderate, low, or very low, with the following domain definitions:  1. Risk of bias: downgrade if high-risk ratings using RoB2 tool. 2. Inconsistency: outcome included 10 or more studies, downgrade if an I^2^ of >50% 3. Indirectness of evidence: study design was for somatostatin use in PLD for primary liver related outcomes.  4. Imprecision of results: downgrade if very wide CIs (level 1) or included the null value (level 2) 5. Publication bias: assessed using funnel plots and Egger’s test for outcomes with 10 or more studies | | | | | | |

Supplementary Table S6. Baseline characteristics of the included patients

| **Study ID** | **Total N** | **Intervention N (% Male)** | **Control N (% Male)** | **Age  mean ± SD** | **TLV (mL/m)   mean ± SD** | **TKV** **(mL/m)   mean ± SD** | **eGFR (mL/min)   mean ± SD** |
| --- | --- | --- | --- | --- | --- | --- | --- |
| **Ruggenenti 2005 [21]** | 12 | 12 (75.0) | 12 (75.0) | Median (range) 44.5 (35–58) | N/A | (Int) 2551±1053  (C) 2461±959 | (Int) 59.5±25.2  (C) 57.9±22.4 |
| **van Keimpema 2009 [22]** | 54 | 27 (11.1) | 27 (14.8) | Mean (95% CI) (Int) 49.6 (34.4–64.8)  (C) 50.3  (32.6–68.1) | Mean (95% CI) (Int) 4606 (547–8665)  (C) 4689 (613–8765) | Mean (95% CI) (Int) 1000  (39 to 2039)  (C) 1115  (519 to 2748) | N/A |
| **Caroli 2010 [23]** | 12 | 12 (75) | 12 (75) | Median (range) 44.5 (35–58) | (Int) 1595 ± 478  (C) 1580 ± 487 | N/A | N/A |
| **Hogan 2010 [24]** | 42 | 28 (17.9) | 14 (7.1) | (Int) 49.7 ± 9 (C) 50.3 ± 7.3 | (Int)5907.7 ± 2915  (C)5373.9 ± 3565 | (Int) 1142.9 ± 826  (C) 803.0 ± 269 | (Int) 70 ± 27  (C) 71 ± 27 |
| **Caroli 2013  (ALADIN) [25]** | 79 | 40 (42.5) | 39 (51.3) | (Int) 36 ± 8 (C) 38 ± 8 | N/A | (Int) 1556 ± 1035  (C) 2161 ± 1274 | (Int) 90 ± 37  (C) 76.1 ± 40 |
| **Pisani 2016 [26]** | 27 | 14 (36) | 13 (38) | (Int) 30 ± 8 (C) 37 ± 8 | (Int) 1609 ± 501  (C) 1693 ± 470 | N/A | N/A |
| **Meijer 2018 [20]  (DIPAK-1)** | 305 | 153 (46.4) | 152 (46.7) | (Int) 48.2 ± 7.4 (C) 48.5 ± 7.2 | N/A | (Int) 2046 ± 1171  (C) 1874 ± 1202 | (Int) 51.0 ± 11.5  (C) 51.4 ± 11.2 |
| **Perico 2019 [27] (ALADIN-2)** | 100 | 51 (60.8) | 49 (53.1) | (Int) 48.7 ± 8.9 (C) 50.0 ± 9.3 | N/A | (Int) 2,338 ± 1362  (C) 2,59 1± 1876 | (Int) 27.9 ± 10.15  (C) 25.8 ± 6.44 |
| **van Aerts 2019 [14]** | 175 | 93 (43.0) | 82 (48.8) | (Int) 48.3 ± 6.2  (C) 48.0 ± 7.0 | (Int) 1528 ± 883  (C) 1376 ± 347 | N/A | N/A |
| **Hogan 2020 [13]** | 48 | 33 (6.1) | 15 (20.0) | (Int) 50 ± 9  (C) 51 ± 8 | (Int) 2582 ± 1381  (C) 2387 ± 759 | (Int) 534 ± 343  (C)397 ± 159 | (Int)74 ± 24  (C)76 ± 17 |
| Abbreviations: TLV, total liver volume; TKV, total kidney volume; eGFR, estimated glomerular filtration rate; mL/m, millilitres per meter; mL/min, millilitres per minute; CI, confidence interval; Int, intervention; C, Control. | | | | | | | |

Supplementary Table S7. Leave-one-out sensitivity analyses for TLV, TKV, and eGFR

| **Study omitted/outcome** | **Effect size** | **(95% CI)** | **I^2^** |
| --- | --- | --- | --- |
| TLV | **Mean difference** |  |  |
| Caroli 2010 [23] | −7.07 | (−9.29 to −4.84) | 0% |
| Hogan 2010 [24] | −6.93 | (−9.16 to −4.71) | 0% |
| Hogan 2020 [13] | −6.26 | (−8.40 to −4.13) | 0% |
| Pisani 2016 [26] | −6.34 | (−8.34 to −4.34) | 0% |
| Van-Aerts 2019 [14] | −7.19 | (−9.63 to −4.76) | 0% |
| Van-Kempen 2009 [22] | −6.85 | (−8.89 to −4.80) | 0% |
| **TKV** | **Mean difference** |  |  |
| Caroli 2013 [25] | −3.43 | (−5.34 to −1.53) | 47% |
| Hogan 2010 [24] | −2.70 | (−3.98 to −1.42) | 20% |
| Hogan 2020 [13] | −3.11 | (−4.80 to −1.42) | 40% |
| Meijer 2018 [20] | −4.16 | (−5.71 to −2.60) | 0% |
| Perico 2019 [27] | −3.52 | (−5.54 to −1.49) | 46% |
| Ruggenenti 2005 [21] | −3.42 | (−4.90 to −1.55) | 48% |
| Van-Keimpema 2009 [22] | −3.41 | (−3.41 to −1.70) | 50% |
| **eGFR** | **Mean difference** |  |  |
| Caroli 2013 [25] | 0.61 | (−5.77 to 6.99) | 61% |
| Hogan 2010 [24] | −0.09 | (−6.05 to 5.87) | 64% |
| Hogan 2020 [13] | 0.15 | (−5.32 to 5.61) | 64% |
| Meijer 2018 [20] | −1.63 | (−6.78 to 3.51) | 28% |
| Perico 2019 [27] | 3.16 | (0.51 to 5.82) | 0% |
| Ruggenenti 2005 [21] | −0.17 | (−5.36 to 5.01) | 63% |
| **Cholelithiasis/cholecystitis** | **Odds ratio** |  |  |
| Caroli 2013 [25] | 3.12 | (0.61 to 16.03) | 0% |
| Meijer 2018 [20] | 6.09 | (1.25 to 29.78) | 0% |
| Perico 2019 [27] | 7.54 | (1.23 to 46.16) | 0% |
| Ruggenenti 2005 [21] | 5.77 | (1.20 to 27.65) | 0% |
| **Abdominal pain** | **Odds ratio** |  |  |
| Caroli 2013 [25] | 6.39 | (1.27 to 32.12) | 87% |
| Hogan 2010 [24] | 6.49 | (1.40 to 30.15) | 87% |
| Meijer 2018 [20] | 3.64 | (1.14 to 11.64) | 62% |
| Perico 2019 [27] | 8.84 | (3.29 to 23.72) | 58% |
| Van-Keimpema 2009 [22] | 4.26 | (1.12 to 16.26) | 87% |
| **Diarrhea** | **Odds ratio** |  |  |
| Caroli 2013 [25] | 14.56 | (2.54 to 83.50) | 88% |
| Hogan 2010 [24] | 15.09 | (2.75 to 82.72) | 88% |
| Meijer 2018 [20] | 5.78 | (3.10 to 10.78) | 0% |
| Perico 2019 [27] | 15.16 | (2.75 to 83.53) | 87% |
| Ruggenenti 2005 [21] | 13.00 | (0.51 to 330.48) | 90% |
| Van-Keimpema 2009 [22] | 10.80 | (1.77 to 65.92) | 90% |
| Abbreviations: TLV, total liver volume; TKV, total kidney volume; eGFR, estimated glomerular filtration rate. | | | |

**
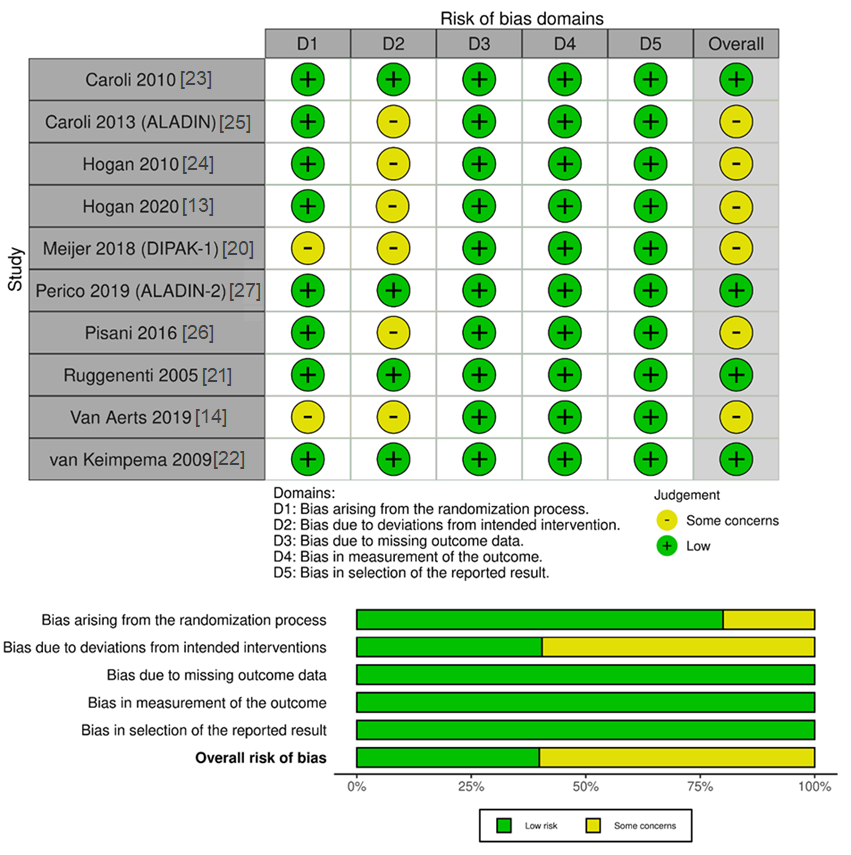
**

Supplementary Figure S1. Risk of bias assessment


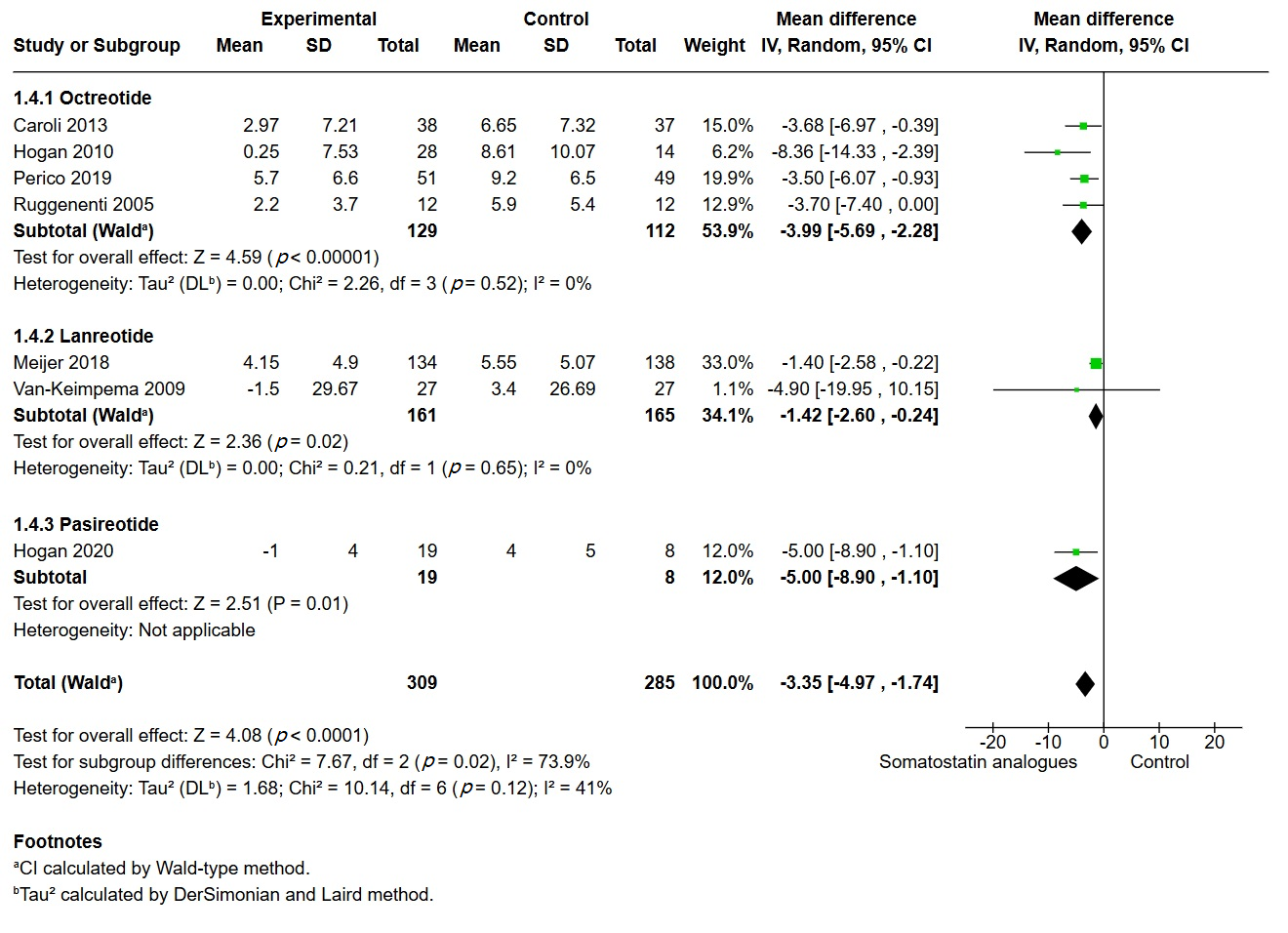


Supplementary Figure S2. Total kidney volume, subgroup analysis


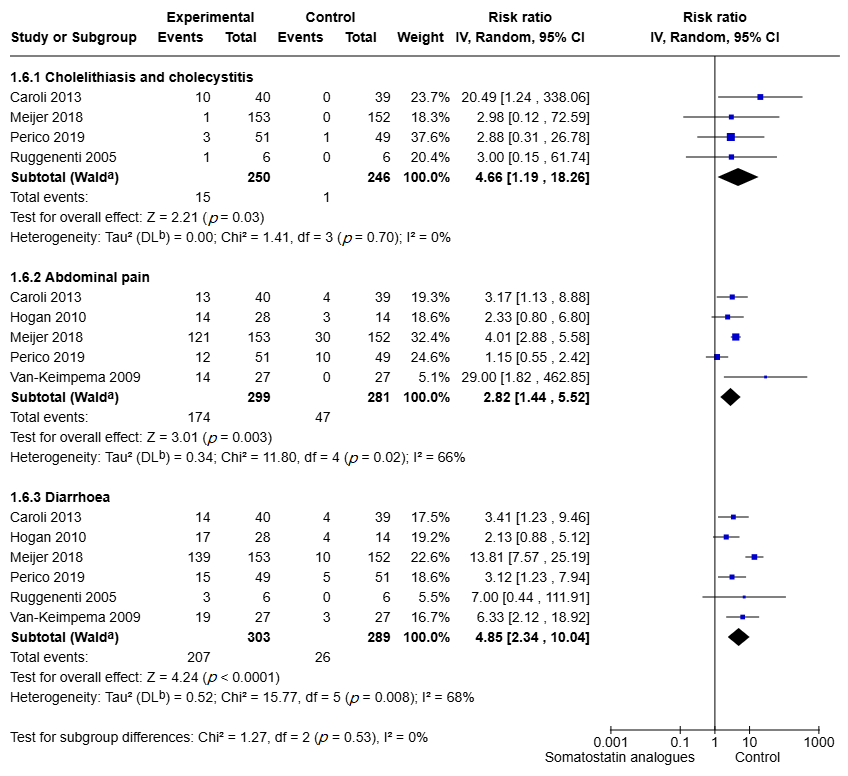


Supplementary Figure S3. Safety outcomes analysis


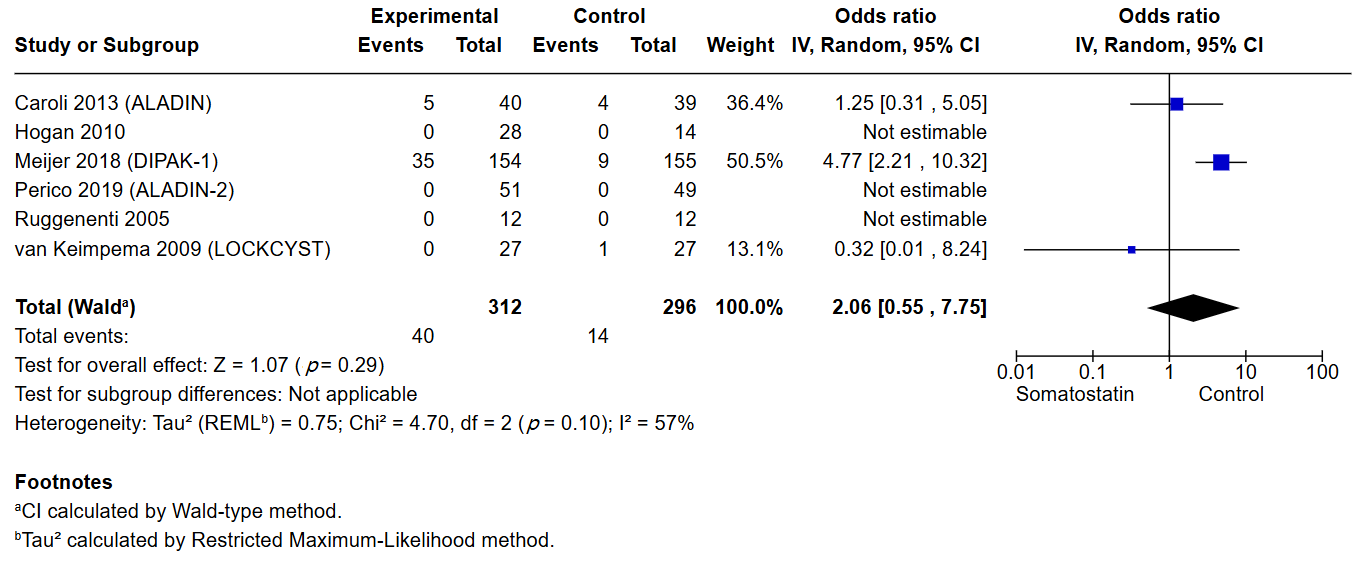


Supplementary Figure S4. Discontinuation rate
